# Topological Data Analysis Identifies Copy Number Variation Subtypes of Bulls Associated with Fertility

**DOI:** 10.3390/ijms27219867

**Published:** 2026-11-04

**Authors:** Siroj Bakoev, Anton Ermilov, Dar’ya Sidorenko, Faridun Bakoev, Olga Kostyunina, Natalia Altukhova, Gleb Nemkovskiy, Vladimir Belyakov

**Affiliations:** 1 Russian State Agrarian University—Moscow Timiryazev Agricultural Academy, 127434 Moscow, Russia; siroj1@yandex.ru (S.B.); sidorenkodaria2000@mail.ru (D.S.); n.altukhova@rgau-msha.ru (N.A.); 2 LLC «WESTTRADE Ltd.», 115201 Moscow, Russia; de_lamak@mail.ru (A.E.); bakoevfaridun@yandex.ru (F.B.); gbnem@mail.ru (G.N.); bel.vk@yandex.ru (V.B.)

**Keywords:** topological data analysis, TDA, Mapper, CNVR, bull fertility, genetic subtypes, structural variation

## Abstract

Bull fertility is a polygenic trait of significant economic importance in dairy and beef cattle, controlled by multiple genetic variants, including copy number variations (CNVs). Conventional single-variant association analyses primarily evaluate marginal effects of individual loci, whereas CNV co-occurrence patterns require complementary approaches. This study applied topological data analysis (TDA) to identify groups of bulls based on genome-wide CNVR profiles and to assess their association with insemination success rate. A total of 88 bulls were included, and the presence/absence of 406 consensus CNVR was determined for each animal. TDA was performed using the Mapper algorithm with a two-dimensional UMAP projection as the lens and DBSCAN for clustering, followed by community detection using the Leiden method. Bootstrap resampling (100 iterations) was used to assess the stability of the identified communities, and their association with fertility was tested using ANOVA and permutation testing (n = 1000). TDA with bootstrap consensus identified five bull communities ranging from 10 to 30 individuals each. The identified subtypes differed in mean insemination success rates, ranging from 48.3% to 72.5%. These differences were assessed using ANOVA and permutation testing. Gene annotation of the loci distinguishing these communities using Ensembl release 110 for the ARS-UCD1.2 assembly revealed genes potentially involved in embryonic development, cellular signaling, and immune response (e.g., *IGF2*, *INS*, *NOTCH1*, *PAEP*, *TRAF2*). TDA provides a framework for identifying groups of bulls with distinct combinations of CNVRs and evaluating their potential association with fertility. Due to the sample size (n = 88) and unadjusted phenotypes, the findings should be considered exploratory and hypothesis-generating. The discovered candidate genomic regions require mandatory validation and independent replication in larger cohorts before being considered for implementation in breeding programs.

## 1. Introduction

Copy number variations (CNVs) represent one of the most significant classes of structural genomic variants, playing a key role in shaping the phenotypic diversity of livestock [1,2,3]. In contrast to single nucleotide polymorphisms (SNPs), CNVs affect a substantially larger proportion of the genome (ranging from 0.13% to 9.42% depending on the species) and are characterized by higher mutation rates—from 1.1 × 10^−2^ to 1 × 10^−8^ per locus per generation [4,5]. This makes CNVs a particularly important source of genetic variation, providing the foundation for both natural and artificial selection. The mechanisms underlying CNV formation include non-homologous end joining (NHEJ), non-allelic homologous recombination (NAHR), and fork stalling and template switching (FoSTeS), which contribute to their high evolutionary dynamics and association with adaptive traits [6,7].

Bull fertility is one of the key economically significant traits in dairy and beef cattle [3,8,9]. Fertility is a complex trait influenced by multiple genetic and environmental factors [2,3,8]. In the context of animal fertility, CNVs are of particular interest, since reproductive traits are characterized by polygenic inheritance and low heritability, which complicates the identification of associated genetic markers [2]. Recent studies demonstrate that CNVs are associated with a wide range of economically important traits, including milk production, growth, disease resistance, and, notably, reproductive performance [3,6,10,11]. For example, a CNV in the KIAA1683 gene on chromosome 7 was identified in the Israeli Holstein population and found to be significantly associated with cow fertility [8]. Similarly, other studies have identified CNV regions associated with semen quality and fertilization capacity in bulls [3,6], opening prospects for marker-assisted selection.

The development of computational approaches, including topological data analysis, provides opportunities for investigating complex genetic data and their relationships with quantitative traits [12,13,14]. These methods complement single-variant analyses by revealing patterns of co-occurrence among genetic features.

Conventional single-variant CNV-GWAS primarily evaluates the marginal effects of individual variants, whereas interactions and patterns of CNV co-occurrence require additional analytical approaches [3,8].

Despite significant progress in CNV detection using NGS technologies and bioinformatics algorithms (read-pair, read-depth, split-read, and assembly-based approaches), unresolved challenges remain, including the analysis of repetitive sequences, regions with high GC content, and low sequencing coverage [1,6]. Furthermore, the functional interpretation of identified CNVs and understanding their role in regulating the expression of genes involved in spermatogenesis, folliculogenesis, and embryonic development remain pressing challenges in modern animal genetics [3,10]. Consequently, there is a need for novel analytical approaches capable of identifying complex, non-linear patterns of association between CNVs and fertility, which motivates interest in topological data analysis (TDA) [12,13].

Topological data analysis (TDA) offers an alternative approach for analyzing complex genetic data [12,13]. In contrast to classical clustering, TDA (specifically, the Mapper algorithm) constructs a graph that reflects the shape of the data distribution in feature space, enabling the identification of groups of objects that are similar in terms of the co-occurrence of CNVs, without a priori assumptions about linearity or additivity of relationships [12]. Topological data analysis represents a mathematical and computational paradigm based on algebraic topology methods, allowing the identification of the internal structure and “shape” of multidimensional data [12,13,14]. The key tools of TDA include persistent homology, which tracks the birth and death of topological features (connected components, loops, voids) across changing scales, and the Mapper algorithm, which constructs a simplified graphical representation of data through clustering and connecting similar objects [12,15]. These methods are model-independent and scale-invariant, making them particularly effective for analyzing complex, non-linear biological data [13,14]. In recent years, TDA has found wide application in biology, including the analysis of tumor heterogeneity, reconstruction of cell differentiation trajectories, identification of rare cell populations, and immune response mapping [15]. For example, in flow cytometry and single-cell RNA sequencing, TDA enables the identification of continuous and branching developmental processes, hidden clusters, and transitional states that elude traditional clustering methods [12,15].

In the context of genetics and animal breeding, applications of TDA remain relatively limited but are actively developing [14]. In the field of three-dimensional genomics, TDA is applied to the analysis of topologically associating domains (TADs) and the study of spatial chromatin organization, which is directly relevant to the regulation of gene expression, including genes involved in reproductive processes [16]. The integration of TDA with machine learning methods (particularly deep neural networks) helps overcome limitations associated with data sparsity and noise, enabling the identification of complex patterns of genome organization [14]. However, to the best of our knowledge, at the time of this study, no research had applied TDA to the analysis of CNV profiles in livestock in the context of fertility [12,13,14,16]. The present study addresses this gap by evaluating the feasibility of using TDA (the Mapper algorithm) to identify groups of bulls based on patterns of CNVR co-occurrence and to investigate their association with insemination success rates.

## 2. Results

### 2.1. CNV and CNVR Identification

PennCNV. PennCNV is a CNV detection algorithm based on the analysis of fluorescence intensity data (log R ratio, LRR) and B allele frequency (BAF) obtained from SNP microarrays. The method employs a hidden Markov model (HMM) that accounts for the spatial dependence of neighboring markers and enables genome segmentation into regions with different copy number states (neutral, loss, duplication). One of the key advantages of PennCNV is its consideration of GC content in genomic regions to correct for systematic signal biases. The algorithm also incorporates mechanisms for filtering noise outliers and assessing the quality of each sample, reducing the likelihood of false-positive detections. The method is widely used in population genetics and medical research to identify rare and common CNVs.

QuantiSNP. QuantiSNP is a CNV detection method that also employs a hidden Markov model but with several statistical differences. Unlike PennCNV, QuantiSNP applies an objective Bayesian approach (objective Bayesian HMM), where model parameters are estimated using non-informative prior distributions. This allows for more flexible adaptation to varying noise levels and marker densities on the microarray. The method computes a Bayes factor for each putative segment, quantitatively assessing the support for the alternative hypothesis (CNV presence) versus the null hypothesis (neutral state). QuantiSNP effectively handles data containing mixed sample populations and provides high sensitivity even with suboptimal data quality. The method also provides probabilistic estimates of CNV boundaries.

GADA. GADA (Genome Alteration Detection Analysis) is a CNV detection method based on sparse Bayesian learning and segmentation of genomic intensity data. In contrast to HMM-based approaches, GADA formulates CNV detection as a problem of partitioning chromosomes into homogeneous segments using L1-regularized regression (LASSO). The method first smooths the LRR signal and then applies an iterative procedure to identify breakpoints by minimizing a penalty function that balances approximation accuracy and model complexity (number of segments). Its segmentation performance depends on marker density, signal quality, and parameter selection, and candidate CNVs require appropriate filtering and validation.

#### 2.1.1. CNV Detection by Three Methods

Analysis of the same set of animals using the three CNV detection algorithms yielded differing results (Table 1). After applying uniform filtering criteria, PennCNV identified 588 CNVs in 83 out of 127 animals, with the majority of events being deletions (84.4%) and a mean CNV length of approximately 625 kb. QuantiSNP detected 2163 CNVs in 121 animals, predominantly duplications (83.7%), with a mean CNV length of 2.04 Mb and a median length of 1.88 Mb. GADA registered the highest number of CNVs (4628) and detected variants in all 127 animals. In contrast to QuantiSNP, GADA was characterized by a pronounced predominance of deletions (94.4%) over duplications (5.6%), with a mean CNV length of 1.90 Mb and a median length of 1.63 Mb.

The obtained results indicate substantial differences in CNV detection profiles among the algorithms. PennCNV was characterized by the most conservative detection of structural variants, identifying fewer events and predominantly shorter CNVs. QuantiSNP detected significantly more variants than PennCNV and was biased toward duplication detection. GADA registered the maximum number of CNVs, with the vast majority of detected events being deletions. After filtering, individual CNV calls ranged from approximately 50–65 kb to 5.0 Mb. The characteristics of the merged CNVRs, which may span larger genomic intervals due to the merging of overlapping calls, are presented separately.

To construct a highly reliable consensus set of CNVs, only those variants that were simultaneously detected by all three methods—PennCNV, QuantiSNP, and GADA—were selected. The intersection reduced method-specific CNV calls, particularly GADA calls not supported by the other algorithms, thereby increasing confidence in the consensus set. The consensus set contains substantially fewer CNVs compared to any of the individual methods; however, it contains CNV calls supported by all three methods. CNVs supported by all three algorithms were considered higher-confidence candidate structural variants, although independent experimental validation is required to confirm their accuracy. This consolidated set was used for subsequent gene annotation and exploratory analyses of associations with phenotypes.

The chromosomal distribution of CNVs differed among the detection algorithms (Figure 1). GADA registered the highest number of CNVs on nearly all autosomes, with the highest concentration of events observed on chromosomes BTA7, BTA11, BTA18, and BTA19. PennCNV identified significantly fewer CNVs, with the most prominent peaks observed on BTA6, BTA7, BTA12, and BTA25. QuantiSNP occupied an intermediate position in terms of the total number of detected variants, characterized by the maximum number of CNVs on BTA6 and BTA12. Despite differences in the absolute number of CNVs detected, all three algorithms demonstrated a similar heterogeneity in the distribution of structural variants across the genome, indicating the presence of chromosomal regions characterized by an elevated concentration of CNVs, regardless of the algorithm used.

Despite differences in the total number of detected CNVs, the distribution of variations across chromosomes was similar in nature. The highest concentration of CNVs was observed on BTA6, BTA7, BTA11, BTA12, and BTA25, whereas the lowest number of events was recorded on BTA13, BTA22, and BTA24. For the GADA algorithm, chromosomes BTA18 and BTA19 were additionally identified, being characterized by an increased density of CNVs.

#### 2.1.2. Merging into CNVR

Analysis of consensus copy number variations (CNVRs) detected simultaneously by three methods (PennCNV, QuantiSNP, and GADA) showed that the total number of consensus regions was 406. The mean CNVR length was 449.5 kb, and the median was 217.5 kb, indicating a wide range of variation sizes—from a minimum of 50.3 kb to a maximum of 32.7 Mb. The considerable gap between the mean and median length points to the presence of a small number of extended CNVRs that increase the mean value. The average population frequency of CNVRs was 12.1%, with a median of 6%, reflecting the predominance of rare variants and indicating heterogeneity of structural variability. The consensus approach yielded 406 candidate CNVRs supported by three detection algorithms, providing a conservative dataset for subsequent exploratory analyses.

Among the 406 consensus CNVRs, the majority were regions with deletions (loss)—360 (88.7%), which is consistent with the overall predominance of losses observed in the individual CNV sets (Table 2).

Duplications (gain) were significantly less common and represented by 31 regions (7.6%). A separate category was formed by 15 CNVRs (3.7%) in which both losses and duplications were detected among different individuals, indicating structural variability in these genomic regions.

Deletion CNVRs were characterized by a mean length of 436.6 kb (median 214.1 kb) and occurred on average in 11.9% of samples. Duplications turned out to be somewhat longer (mean length 630.7 kb) and, as a rule, demonstrated a lower population frequency (7.3%). The most interesting group is represented by CNVRs with a mixed type (both), which not only had an intermediate length (384.2 kb) but also a significantly higher occurrence frequency (28.9%), suggesting the presence of both deletions and duplications in these regions in different individuals, as well as their potential functional significance.

Analysis of the distribution of CNVRs across chromosomes depending on the variant type showed that deletions (loss) are the dominant type on all 29 autosomes (Figure 2). The largest number of deletion CNVRs was found on chromosomes BTA1 (25), BTA2 (22), BTA4 (21), BTA6 (23), BTA7 (30), BTA8 (22), BTA11 (24), BTA12 (27), and BTA19 (21). The minimum number of losses was recorded on BTA22 (6), BTA24 (8), and BTA28 (9). Duplications (gain) occurred much less frequently and were distributed unevenly: the maximum was noted on BTA6 (5), BTA7 (4), and BTA12 (5), whereas most chromosomes had from 0 to 3 events. Mixed variants (both) turned out to be the least common and were localized predominantly on BTA2, BTA3, BTA4, BTA6, BTA7, BTA8, BTA12, BTA13, BTA15, BTA18, and BTA19, with no more than 1–2 such regions identified on each of them.

The obtained results indicate uneven accumulation of different types of CNVRs across the cattle genome. Chromosomes BTA6, BTA7, and BTA12 turned out to be the most variable with respect to all types of structural changes, which is consistent with previously identified CNV “hotspots.” The predominance of deletions on most chromosomes may reflect both biological features (greater tolerance to loss of genetic material at certain loci) and technical characteristics of the detection methods. The low frequency of duplications and mixed variants is probably due to their lower prevalence in the population or to more complex algorithmic detection.

### 2.2. Stable Communities of Bulls Based on CNVR Profiles

Topological analysis (Mapper + Leiden + bootstrap consensus) identified five communities of bulls based on their CNVR profiles (Figure 3).

The size of the communities varied from 10 to 30 individuals (Table 3). Six bulls (Bull_20, Bull_32, Bull_46, Bull_49, Bull_81, Bull_86) were represented in Mapper nodes assigned to different communities. For subsequent statistical analyses, each bull was assigned to a single community using the final consensus partition, ensuring non-overlapping groups.

### 2.3. Association of Communities with Fertility

The communities differed significantly in fertilization rate (ANOVA: F = 11.68, *p* = 1.42 × 10^−7^). The highest fertility was observed in Community 4 (72.5 ± 10.2%), and the lowest in Community 2 (48.3 ± 11.8%). Figure 4 demonstrates the distribution of fertilization rate among the five stable genetic subtypes of bulls identified using topological data analysis (TDA). In panel A (boxplot), it is clearly visible that Community 4 is characterized by the highest median fertility (~73%), whereas Community 2 has the lowest (~48%). Community 0 occupies an intermediate position (~68%), while Communities 1 and 3 cluster around 52–55%. The global ANOVA was used to assess phenotypic differences between communities, providing an initial evaluation of the relationship between CNVR-based population structure and insemination success rate.

In panel B (kernel density estimates), differences in the shape of the distributions draw attention. Community 4 showed a relatively narrow phenotypic distribution, suggesting lower within-group variability in insemination success rate. However, this pattern should be interpreted cautiously because environmental and management-related factors were not included in the analysis. In contrast, Community 1 showed greater phenotypic variability, while Community 3 exhibited an apparently bimodal distribution. The factors underlying these patterns remain unknown.

The most pronounced differences were observed for CNVRs on chromosome 6 (C): in Community 4, the frequency of these CNVRs reached 94–100%, whereas in Community 3 they were completely absent (0%). Similarly, CNVRs on chromosome 21 (BTA21:70393623-71136925) were almost universal in Community 4 (100%), but absent in Community 1 (6%) and Community 3 (0%).

A permutation test (n = 1000) was performed to assess the significance of the observed association. The empirical *p*-value was calculated by comparing the observed ANOVA F-statistic with the permutation distribution, using a finite-sample correction. This test evaluates phenotype–community association rather than the stability of community assignments.

The obtained patterns are consistent with the data on the CNVR profiles of the communities. Community 4, with the highest mean insemination success rate, shows high frequencies of CNVRs on chromosomes BTA6, BTA7, and BTA12, whereas Community 2, with the lowest mean rate, shows CNVRs on chromosomes BTA7, BTA6, and BTA4, with lower frequencies of CNVRs on chromosome 12. The relatively narrow phenotypic distribution of Community 4 suggests that its characteristic combination of CNVRs may be associated with a more homogeneous fertility profile, a hypothesis that requires further validation. At the same time, the broader variability of Community 1 and the apparent bimodality of Community 3 could reflect heterogeneous factors that were not evaluated in this study. These observations support the use of TDA as an exploratory approach for investigating heterogeneous genetic data structures that may be difficult to characterize using some conventional clustering methods.

### 2.4. Frequently Detected CNVRs in the Phenotyped Cohort

To provide a reproducible description of the updated catalog, CNVRs were ranked by the number of positively detected carriers among the 88 phenotyped bulls. Table 4 reports the exact ARS-UCD1.2 coordinates, variant direction, and total observed carrier counts for the five most prevalent structural variants in our cohort. Consistent with our exploratory framework, phenotype effect estimates or association *p*-values are not assigned to these individual regions without further validation.

### 2.5. Gene Annotation of CNVRs

For the functional interpretation of the identified CNVRs, we performed their annotation strictly within the Bos taurus genome ARS-UCD1.2 assembly space using Ensembl release 110 to avoid coordinate mismatch. Distinct gene overlap profiles were identified for the CNVRs characterizing each community (Table 5).

CNVRs enriched in communities with higher mean insemination success rates showed a higher average GC content (56.2%) than those enriched in communities with lower mean rates (40.9%). Given the higher GC content often observed in coding and regulatory regions, the detected differences may reflect variation in the genomic context of CNVRs across communities. Gene overlap and differences in GC content do not establish functional consequences. Further functional analyses are required to determine whether the identified CNVRs affect gene regulation or expression.

## 3. Discussion

### 3.1. TDA as a Hypothesis Generator

In this work, we demonstrated that TDA (Mapper + Leiden) allows the identification of groups of bulls based on combinatorial CNVR profiles. It is important to emphasize that TDA is not an association method—it does not compute the relationship with a phenotype, but merely reveals the internal topological structure of multidimensional data based on the co-occurrence of features. This is a fundamental difference from classical statistical approaches, which are aimed at testing a priori hypotheses about the relationship between variables. TDA, by contrast, acts as a hypothesis generator: the relationship between community membership, CNVR frequencies, and phenotypic variation was then examined using statistical tests (ANOVA, Fisher’s exact test, and *t*-tests). Such an approach is especially valuable in situations where the researcher has no a priori assumptions about the structure of the data or when the presumed relationships are nonlinear or combinatorial in nature.

This two-stage approach (exploratory TDA → association analysis) facilitates the identification of complex CNVR co-occurrence patterns that may not be readily captured by conventional single-variant GWAS approaches. In our study, these patterns distinguished groups of bulls with different mean insemination success rates, providing candidate genomic profiles for further investigation. Moreover, TDA does not require a priori specification of the number of clusters and takes into account the possibility of overlap between groups, which allowed us to identify borderline bulls with ambiguous membership—a feature that may be less apparent in clustering approaches requiring exclusive assignment of each individual to a single group. Thus, the proposed approach does not replace but effectively complements classical association analysis, providing the first, exploratory stage in the study of polygenic traits.

In the context of animal husbandry and agricultural animal genetics, the application of TDA is at an early stage, but the prospects of this approach appear significant [14]. Modern research in the field of cattle genomics faces the problem of analyzing high-dimensional data (p >> n) and the need to account for epistatic interactions. TDA offers a computationally efficient solution for the visualization and clustering of such data, allowing the researcher to “see” the shape of the distribution of genetic variants in multidimensional space. In the present work, we applied TDA for the first time to analyze CNVR profiles of bulls in the context of fertility. The obtained results suggest that this method can identify distinct CNVR-based groups of bulls that may differ in an economically important reproductive trait—insemination success rate. This opens new opportunities for the application of TDA in breeding programs, in particular for stratifying animals by genetic subtypes before conducting GWAS, for selecting candidate markers, and for identifying individuals with intermediate, “hybrid” genotypes.

### 3.2. Functional Annotation of CNVRs

#### 3.2.1. CNVRs Characterizing High-Fertility Communities

By strictly aligning our consensus CNVRs to the Ensembl release 110 (ARS-UCD1.2) annotation, we identified several regions overlapping protein-coding genes with plausible biological roles in development and cellular signaling. For instance, community 0 was characterized by CNVRs overlapping the IGF2-INS-TH gene cluster on BTA29 and the NOTCH1 and PAEP genes on BTA11. IGF2 (Insulin-like growth factor 2) is a well-characterized imprinted gene that plays a fundamental role in embryonic and fetal development in cattle, making it an intriguing candidate for reproductive traits. PAEP (Progestagen-associated endometrial protein) is also widely known for its critical role in modulating the reproductive immune response. Additionally, Community 4 was characterized by CNVRs on BTA12 overlapping LAMP1 and GAS6, genes associated with lysosomal function and cell survival.

#### 3.2.2. CNVRs Characterizing Intermediate and Low-Fertility Communities

Conversely, Community 1 was characterized by structural variations on BTA11 overlapping TRAF2, a gene heavily involved in cellular stress responses. Interestingly, Community 3 was topologically distinguished not by the presence of specific CNVRs, but rather by the significant absence of the major structural variants prevalent in the rest of the herd. This community may represent a relatively CNVR-depleted profile within the analyzed dataset.

#### 3.2.3. Hypothesis Generation and the Need for Validation

Although the identified CNVR patterns distinguish the topological communities, their relationship with fertility and the functional relevance of overlapping genes require further validation. The presence of a gene within a CNVR boundary does not automatically equate to altered gene expression or a direct phenotypic consequence. As our study was conducted on a limited cohort of 88 bulls and the primary phenotypic trait was unadjusted for environmental covariates, these findings serve as a hypothesis generator rather than definitively proven diagnostic tools. The highlighted CNVRs and their overlapping genes (e.g., IGF2, NOTCH1) represent candidate genomic regions for further investigation and require independent validation in larger cohorts.

### 3.3. Practical Recommendations

The proposed analytical approach can be used in future genetic research for:

• Stratification of bulls by genetic subtypes before association analysis;
• Selection of candidate CNVRs for validation in independent samples;
• Identification of borderline individuals with intermediate CNVR profiles.

### 3.4. Comparison with Existing CNV Catalogs and Methodological Context

To contextualize our findings, we compared the CNVRs identified in this study with those previously reported in Holstein cattle using the CNV catalog of Delledonne et al. [17], which includes 123,814 CNVs detected in 3809 Holstein cows genotyped with the GGP Bovine 100K array and merged into 1397 CNVRs. Our CNVR coordinates were converted from UMD3.1 to ARS-UCD1.2 with the UCSC liftOver tool, and 377 of 406 CNVRs were successfully converted. In total, 232 CNVRs (61.5%) overlapped at least one CNVR reported by [17], compared with 93.5 expected by chance (2.48-fold enrichment, *p* < 0.001, 1000 permutations). The overlapped CNVRs corresponded to 143 of the 1397 reference regions. For the frequent reference CNVRs, carried by ≥2% of cows, 81 of our CNVRs (21.5%) overlapped (3.25-fold enrichment, *p* < 0.001). The overlap rate was higher for CNVRs carried by at least two bulls (192 of 277; 69.3%) than for singletons (40 of 100; 40.0%). Overall, 82.4% of all CNV occurrences (bull × CNVR) fell within confirmed regions.

Notably, a reciprocal overlap of ≥50% was observed for only 12.2% of CNVRs. This indicates that the agreement between studies mainly concerns shared genomic regions rather than identical CNV boundaries. Such a pattern is expected and highlights the inherent methodological differences between the studies, including the sex of the animals, sample size, genotyping arrays (e.g., SNP50K in our study vs. GGP Bovine 100K in the reference), and the specific CNV calling algorithms employed. This comparison reinforces the reliability of our consensus CNVR set while acknowledging the well-known challenges in precisely defining structural variant boundaries across different genomic platforms (see Supplementary Table S1 and S2 for detailed overlap metrics per CNVR and the list of non-converted regions, respectively).

## 4. Materials and Methods

### 4.1. Data

The study included 127 Holstein bulls from JSC “Moskovskoe” for Breeding Work (Moscow Region), from which blood was collected and preserved in K3EDTA. All bulls were maintained under identical housing, feeding, and management conditions and received the same diet. Whole-genome genotyping was performed at the genomic selection center of Miratorg-Genetics Center for Genomic Selection, Domodedovo, Moscow Region, Russia” using the Bovine SNP50V3 Illumina, Inc., San Diego, CA, USA medium-density chip in accordance with the manufacturer’s protocol on DNA samples with a concentration of at least 20 ng/µL and an A230/260 ratio of 1.8–2.0.

To assess the reproductive ability of bulls, the main phenotypic trait used was the artificial insemination success rate (Success%), defined as the proportion of inseminations that resulted in pregnancy out of the total number of inseminations performed. Despite the influence of a number of factors related to the condition of the cows and insemination conditions, this indicator is widely used to characterize bull fertility under production conditions, since it integrally reflects their ability to ensure the onset of pregnancy. Unlike absolute indicators such as sperm count or ejaculate volume, insemination success rate directly characterizes the final efficiency of herd reproduction, which makes it the most relevant for breeding and veterinary tasks.

Insemination success rates were initially evaluated separately for cows and heifers to account for differences associated with physiological status. For the present CNVR-based analysis, only insemination success rates recorded in cows were considered. To ensure the statistical reliability of the phenotype and account for the variation in the number of inseminations per bull, the analysis included only animals with a sufficiently large and representative history of recorded inseminations, minimizing potential biases. The final phenotypic trait used for the 88 bulls was the insemination success rate in cows (continuous variable; range: 5.79–89.1%).

### 4.2. CNV Detection

To identify copy number variations (CNVs) in 127 bulls, three independent algorithms were used: PennCNV [18], QuantiSNP [19], and GADA [20]. In the case of PennCNV, GC-content correction was preliminarily performed to eliminate the “genomic wave” artifact. For the Bos taurus ARS-UCD1.2 (bosTau9) assembly, based on the reference genome downloaded from the UCSC Genome Browser, the GC percentage was calculated for all autosomes using a sliding window (size 1000 bp, step 500 bp), forming a tab-delimited file with the columns “chromosome, position, % GC.” The resulting GC file was used with the cal_gc_snp.pl script from the PennCNV package to estimate the GC content around SNP markers. CNV detection was performed with the detect_cnv.pl command with the following parameters: HMM hhall.hmm, PFB file, GC model, logging, and output raw file. Samples were excluded if they met any of the following quality-control criteria: LRR_SD ≥ 0.3, BAF_DRIFT ≥ 0.02, |WF| ≥ 0.05, or 50 or more CNV calls per sample. Individual CNV calls were retained only if they contained at least three SNPs and were at least 50 kb in length. QuantiSNP2 was run in the Windows environment with a command specifying chromosomes (1–29), level files, the configuration file params.dat, and input data; only .cnv files were selected from the output files, which were merged in Linux with the cat command, after which records with a Bayesian coefficient ≤ 10 were removed. For GADA in the R environment, the libraries gada, data.table, stringi, plotrix, and GenomicRanges were loaded; for each sample, Illumina data were configured (log2ratio column = 6), then the SBL steps (estim.sigma2 = TRUE, aAlpha = 0.8) and BackwardElimination (T = 6, MinSegLen = 3) were applied sequentially. Copy-number-neutral segments and segments on chromosomes X and Y were excluded from the resulting summary; the state-code mapping used for filtering requires confirmation against the GADA output. The results for each sample were saved in text files.snp.plcnv.plcnv.plsnp.plcnv.plcnv.pl

For each of the 127 bulls, sets of CNVs detected by three programs (PennCNV, QuantiSNP, GADA) were obtained. All individual CNV calls were combined into common lists separately for each method. The intersection of the results of the three algorithms and the identification of copy number variation regions (CNVRs) were performed using the CNVRanger package in the R environment. Of the 127 initial samples, 88 bulls were included in the analysis after consensus filtering. Overlapping regions of CNVs from different methods were combined into consensus CNVRs, which made it possible to minimize the number of false-positive detections and obtain a high-confidence set of copy number variations for further analysis. For further analysis, a dataset was formed containing animal IDs, the phenotype, and a binary CNVR presence (1)/absence (0) matrix. The input feature set for Mapper should be verified to contain only CNVR variables. The number of columns with CNVRs was 406 CNVRs.

### 4.3. Topological Data Analysis (TDA)

TDA was performed using the kmapper library (Python). The Mapper algorithm [21] was implemented with the following parameters: A two-dimensional UMAP projection was used as the Mapper lens. To assess the influence of the key hyperparameters of the Mapper algorithm on the structure of the resulting graph, a sensitivity analysis was performed. Three parameters were varied: the number of coverage intervals (n_cubes = 6, 8, 10, 12), the degree of interval overlap (perc_overlap = 0.2, 0.3, 0.5), and the eps parameter of the DBSCAN algorithm used for clustering within coverage cells (eps = 0.5, 1, 1.5). For each combination of parameters, a Mapper graph was constructed and the following characteristics were calculated: total number of nodes, number of edges, and number of connected components. The results were saved for subsequent comparison and selection of a stable configuration. As a result, the following were obtained: number of intervals: n_cubes = 10; overlap: overlap_perc = 0.5; clusterer: DBSCAN (eps = 0.5, min_samples = 2). For each bull, a set of Mapper graph nodes into which it falls was determined.

### 4.4. Community Detection and Bootstrap Consensus

The Leiden algorithm (leidenalg implementation, resolution parameter 1.0) was applied to the Mapper graph. To assess the robustness of the identified communities, bootstrap was performed (100 iterations): at each iteration, a random sample with replacement (n = 88) was constructed, the Mapper + Leiden process was repeated, and community labels were recorded for each bull.

Based on 100 bootstrap iterations, an 88 × 88 consensus matrix was constructed to summarize joint community membership across resampled datasets. The final consensus partition into 5 communities was obtained by spectral clustering of this matrix.

### 4.5. Association Analysis

All tests were performed after fixing the consensus communities. The association of communities with fertility was tested using one-way analysis of variance (ANOVA) followed by pairwise *t*-tests with Bonferroni correction (α = 0.05/10 = 0.005). Search for CNVRs characteristic of a community: for each community c and each CNVR, a two-sided Fisher’s exact test was applied (comparison of the CNVR frequency within the community vs. all others). Correction for multiple comparisons—FDR (Benjamini–Hochberg, q < 0.05). Search for CNVRs associated with fertility: for each CNVR, groups of bulls “with CNVR” and “without CNVR” were compared using a *t*-test (with a minimum group size ≥3). Correction—FDR (q < 0.05).

### 4.6. Gene Annotation of CNVRs

For the functional interpretation of the identified CNVRs, annotation was performed using the Bos taurus genome (bosTau9/ARS-UCD1.2 assembly) and the Ensembl database (release 110). Both CNVRs and gene coordinates were strictly matched within the ARS-UCD1.2 assembly space using GenomicRanges to avoid systematic coordinate displacement.

### 4.7. Permutation Test

To assess the probability of obtaining a significant association of communities with fertility on random data, permutation was performed (1000 iterations): the phenotype y was randomly shuffled, and ANOVA was repeated for the fixed consensus communities. The empirical *p*-value was calculated as (b + 1)/(B + 1), where b denotes the number of permutations yielding an ANOVA F-statistic at least as large as the observed statistic, and B = 1000.

### 4.8. Software

All computations were performed in the R environment (v.4.2) [22] and Python (v.3.10) [23] using the packages tidyverse [24], ggplot2 [25], leidenalg [26], scipy [27], statsmodels [28], BSgenome.Btaurus.UCSC.bosTau9 [29], AnnotationHub [30], GenomicRanges [31]. The code is available upon request.

## 5. Conclusions

In the present study, copy number variation (CNV) detection was performed using three independent algorithms—PennCNV, QuantiSNP, and GADA—which made it possible to increase the reliability of identifying candidate CNVs through the intersection of results from different methods. To correct the “genomic wave” artifact arising from variability in GC content in the cattle genome, GC-content correction was applied during PennCNV analysis to reduce potential systematic biases in CNV detection. Based on consensus CNVs detected simultaneously by all three methods, a binary presence/absence matrix of 406 CNVRs was generated for 88 bulls. Topological data analysis (TDA) in combination with bootstrap consensus allowed the identification of five CNVR-based groups of bulls based on the analysis of the obtained CNVR markers. The communities differed in mean insemination success rates; an ANOVA reconstructed from the reported group summary statistics yielded F = 11.68 and *p* = 1.42 × 10^−7^. These estimates require verification against the underlying individual-level data. CNVR patterns distinguishing the communities provide candidate genomic regions for further investigation. Gene annotation identified CNVRs overlapping genes involved in developmental processes, cellular signaling, and immune regulation, providing candidate genomic regions for further investigation of reproductive traits.

It is important to emphasize that TDA is not intended to replace classical association analysis, but rather acts as a tool for generating hypotheses about the combinatorial and potentially nonlinear structure of genetic data. The use of a consensus approach to CNV detection (PennCNV, QuantiSNP, GADA) made it possible to minimize the number of false-positive calls characteristic of each method individually, and GC-content correction helped reduce the influence of systematic technical artifacts. The results obtained demonstrate that the use of TDA as the first stage of analysis allows the identification of hidden patterns of CNVR co-occurrence that may remain unnoticed when using traditional approaches. On this basis, it appears appropriate to recommend a two-stage approach (exploratory TDA → confirmatory classical association analysis) for studying polygenic traits, especially under conditions where the number of features significantly exceeds the sample size (*p* >> n).

Previous studies have shown that structural variations in cattle can be associated with reproductive performance [9]. Our updated annotation suggests that combinatorial patterns of CNVRs on chromosomes such as BTA11, BTA12, and BTA29 may contribute to distinguishing bulls with different topological profiles. This observation is consistent with the hypothesis that reproductive traits may be controlled not so much by individual genetic variants as by potential interactions among multiple genetic variants. The use of three different CNV detection algorithms increases the reliability of the identified genetic variants, which is especially important when working with a limited-size sample, where each false-positive signal can substantially affect subsequent conclusions. Thus, the results of the present study contribute to the understanding of the genetic architecture of fertility and open prospects for further study of epistatic effects using a combination of structural variant detection methods and topological data analysis.

Despite the results obtained, it is necessary to acknowledge the presence of certain limitations. The relatively small sample size (n = 88) may limit the statistical power of the study, and the identified associations require confirmation in independent cohorts. In addition, the functional significance of the identified CNVRs and their presumed influence on the expression of neighboring genes require experimental validation using functional genomics methods. It should also be taken into account that CNV detection from SNP-chip data is associated with certain methodological difficulties, including ambiguous identification of variation boundaries and different sensitivity of the algorithms used to CNVs of different sizes. Nevertheless, the presented approach, combining consensus CNV detection with topological data analysis, demonstrates the potential of TDA as an additional tool in the arsenal of methods for analyzing high-dimensional genetic data, which may contribute to a deeper understanding of the genetic basis of complex traits in farm animals. These findings remain exploratory and require independent validation in larger cohorts before practical implementation in breeding programs.

## Figures and Tables

**Figure 1:**
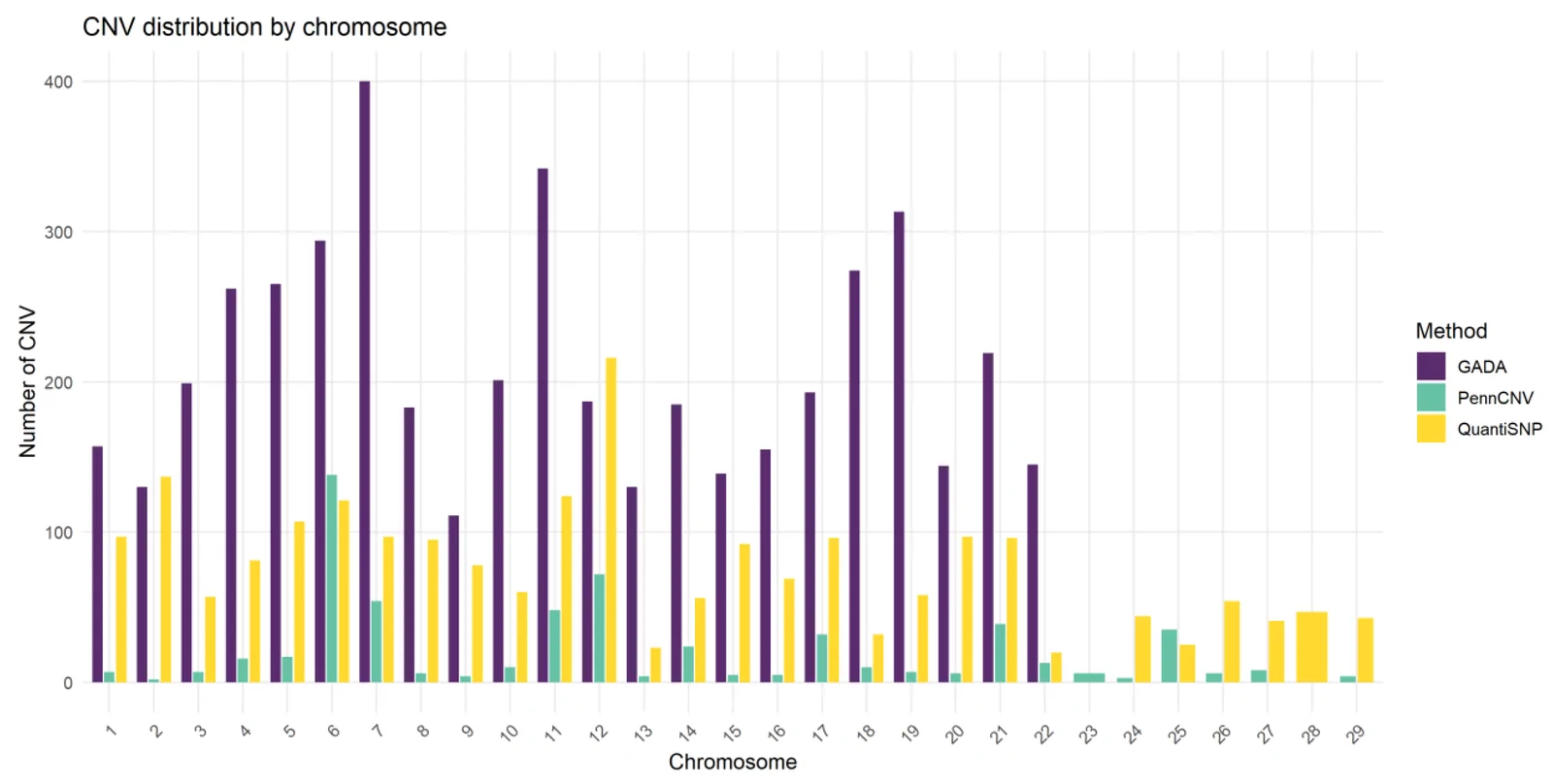
Distribution of CNVs found by three methods across chromosomes.

**Figure 2:**
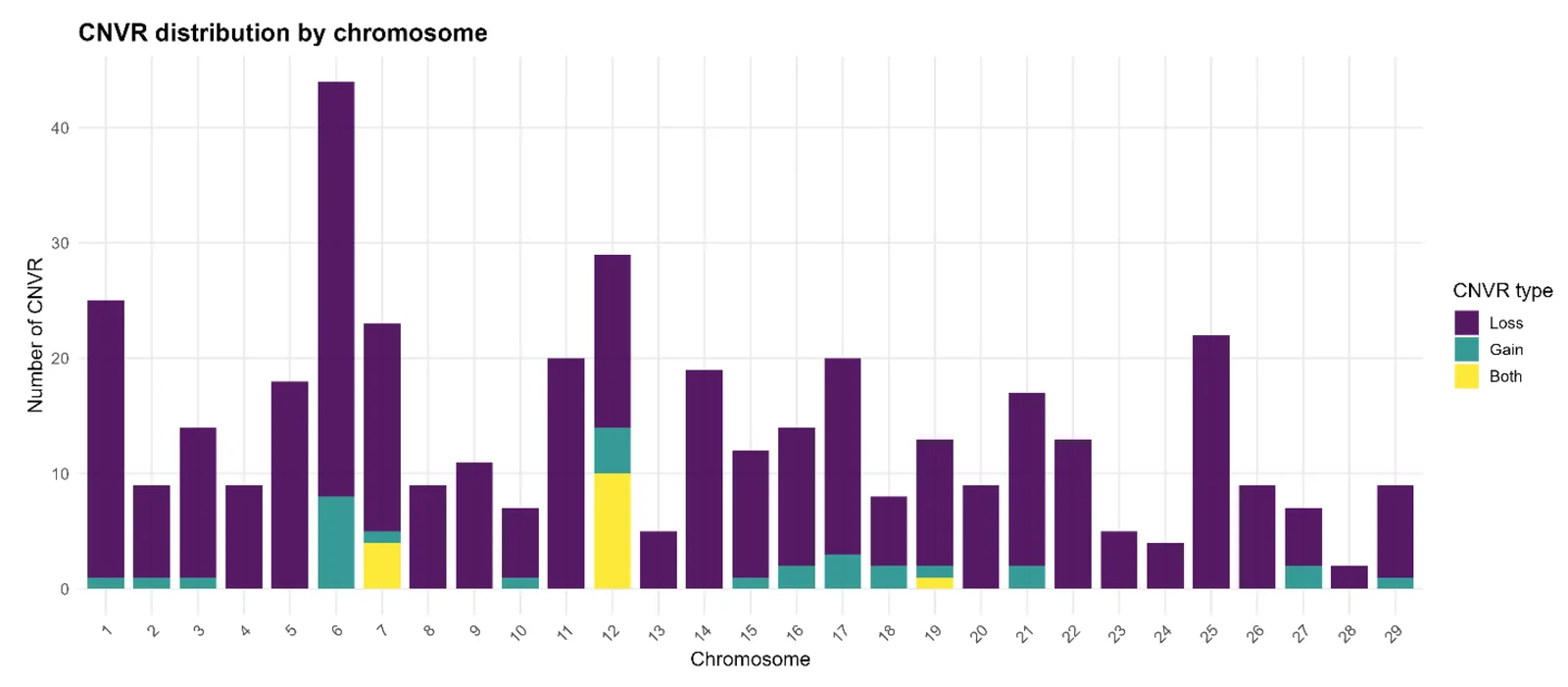
Distribution of consensus CNVRs of different types across chromosomes.

**Figure 3:**
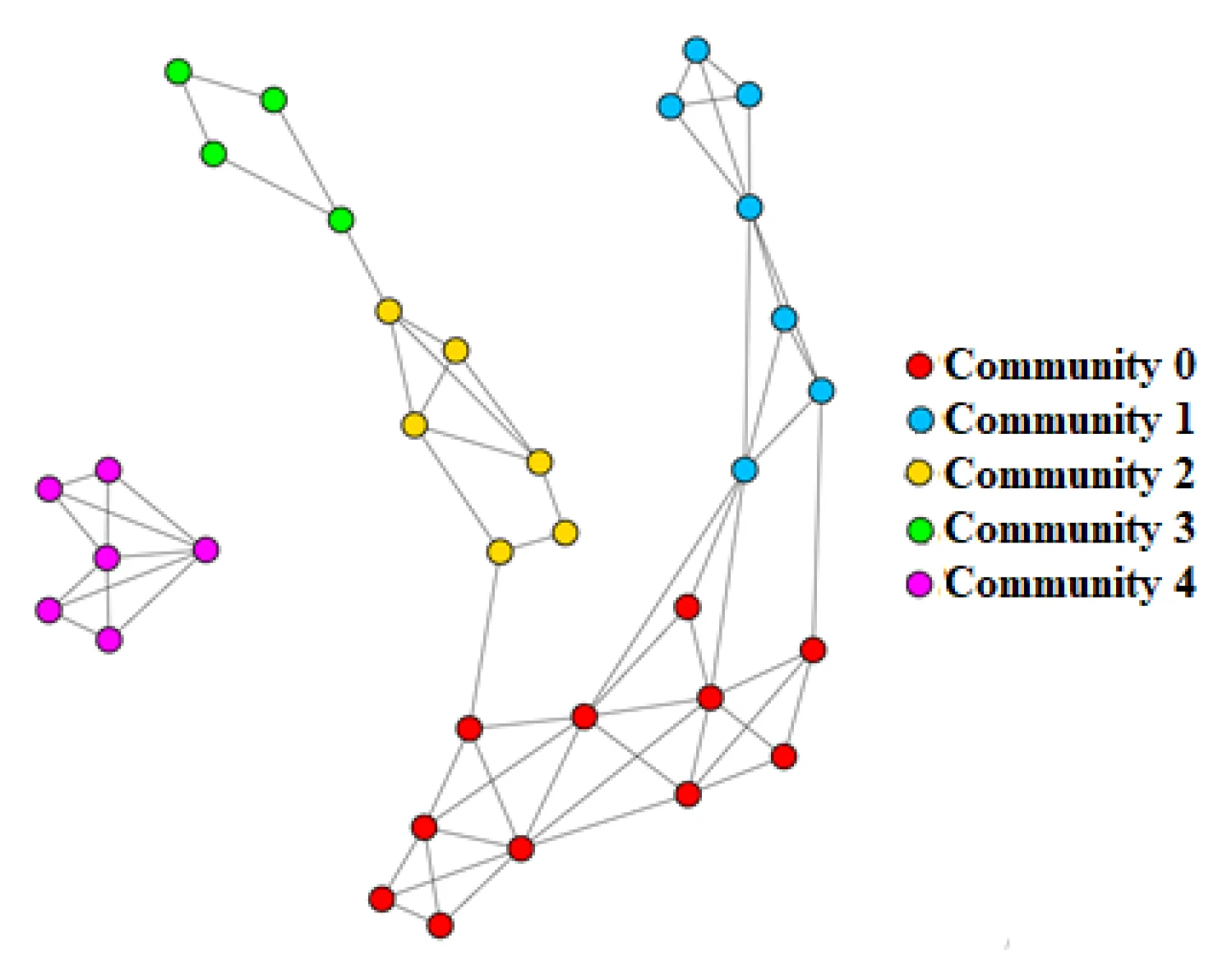
Graph obtained as a result of applying Mapper and community partitioning using the Leiden algorithm.

**Figure 4:**
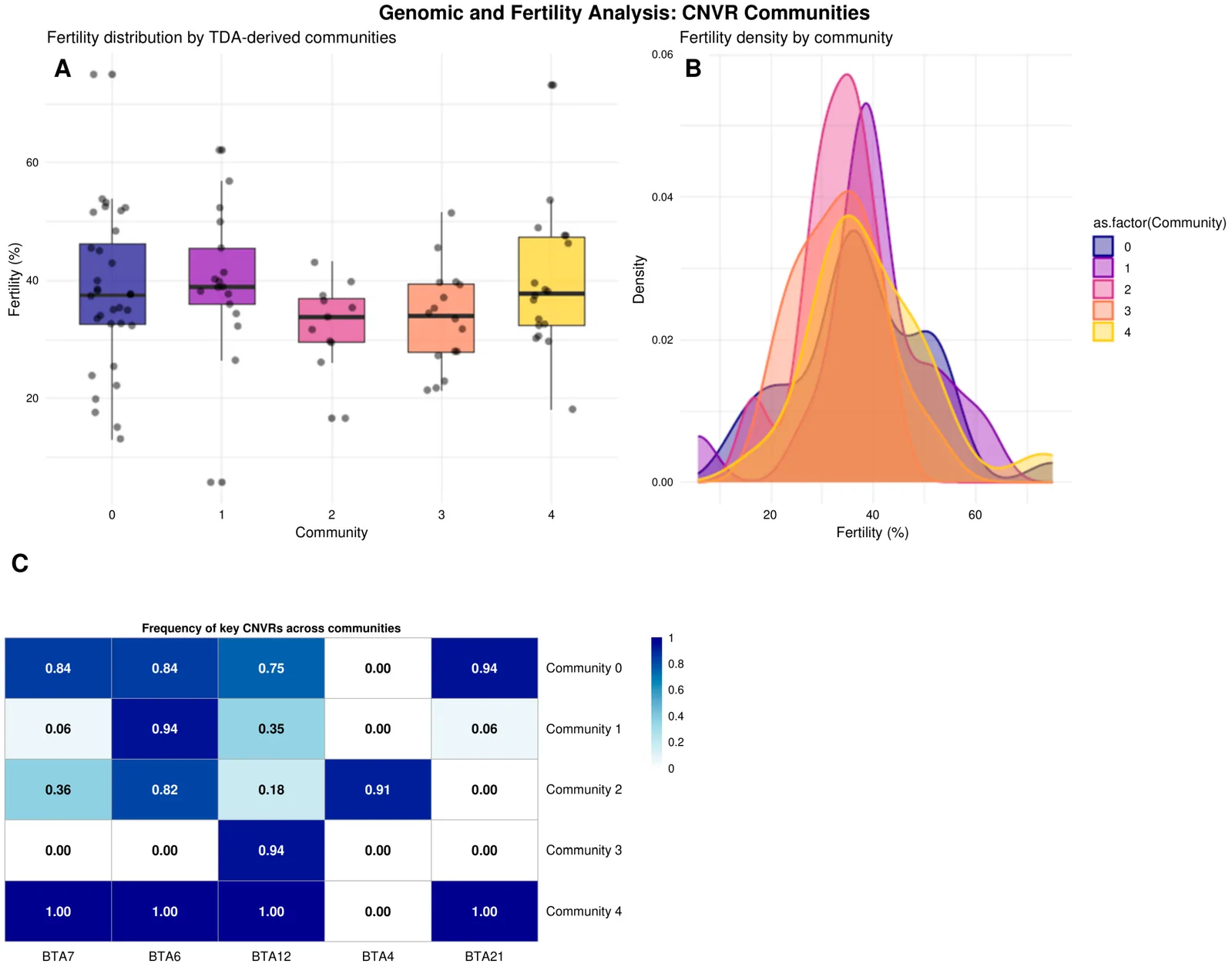
Distribution of fertility across five TDA communities of bulls. (**A**) Boxplot of fertilization rate by community; (**B**) Kernel density estimates of fertilization rate by community; (**C**) Frequencies of the most discriminant CNVRs across communities.

**Table 1:** Summary of CNVs detected by PennCNV, QuantiSNP, and GADA algorithms.

Method	Total CNV	Samples	Mean Length bp	Median Length bp	Min Length bp	Max Length bp	Gain Count	Gain Pct	Loss Count	Loss Pct
PennCNV	588	83	624,644	508,883	54,134	3,911,421	92	15.6	496	84.4
QuantiSNP	2163	121	2,041,333	1,884,194	65,198	4,997,880	1810	83.7	353	16.3
GADA	4628	127	1,896,981	1,627,781	50,863	4,999,214	259	5.6	4369	94.4

**Table 2:** Characteristics of consensus CNVRs by types of structural variants.

Type	CNVR_Count	Percent	Mean Length kb	Median Length kb	Mean Frequency	Median Frequency
loss	360	88.67	436.58	214.06	11.86	6
gain	31	7.64	630.7	235.04	7.32	3
both	15	3.69	384.22	321.13	28.93	34

**Table 3:** Characteristics of communities.

Community	Number of Bulls (N)	Mean Fertility (%)	SD	Min	Max
0	30	68.2	12.4	41.2	88.5
1	16	54.7	14.1	32.4	79.3
2	10	48.3	11.8	27.3	71.6
3	15	51.9	13.5	22.9	75.8
4	17	72.5	10.2	45.6	89.1

**Table 4:** Five CNVRs with the largest detected carrier counts among the 88 phenotyped bulls.

CNVR	Coordinates ARS-UCD1.2	Type	Total Detected Carriers
CNVR00059 *	BTA26:25632934-25755158	loss	28
CNVR00058 *	BTA26:25632934-25700527	loss	25
CNVR00015	BTA6:51924818-52198139	loss	16
CNVR00014	BTA6:37391915-37505093	loss	10
CNVR00064	BTA29:49378009-49601476	loss	8

* Note: CNVR00058 and CNVR00059 represent overlapping genomic intervals on BTA26. These regions were retained as separate records in the CNVR catalog and may therefore represent correlated features in the binary CNVR matrix.

**Table 5:** Descriptive gene annotation of CNVRs enriched in each topological community (ARS-UCD1.2 assembly).

Community	CNVRs with Genes *	Unique Genes *	Examples of Overlapping Genes Characterizing the Community
0	13	93	*IGF2*, *INS*, *TH*, *NOTCH1*, *PAEP*, *KCNT1*, *CAMSAP1*, *MAD1L1*, *GNA12*
1	3	19	*TRAF2*, *PTGDS*, *LCN12*, *FBXW5*, *C8G*
2	3	13	*PCDHAC2*, *PCDHB1*, *MIF*, *SUSD2*, *GGT1*, *UPB1*, *BCR*
3	0	0	*(Characterized by a lower representation of major CNVRs observed in other communities)*
4	12	59	*LAMP1*, *GRK1*, *GAS6*, *TFDP1*, *TMCO3*, *TACC3*, *SLBP*

* Note: Gene counts represent the output of the annotation pipeline for CNVRs enriched in the respective community. Community 3 is topologically distinguished by the lack of the major structural variants prevalent in other groups.

## Data Availability

Due to commercial non-disclosure agreements, raw SNP array intensity data (LRR and BAF files) are not publicly available. To support the reliability of the consensus CNVR set identified in this study, a detailed comparative analysis with the independent CNV catalog of [17] is provided in Supplementary Table S2, including summary confirmation rates, the row-by-row comparison dataset, and the list of regions that could not be converted from UMD3.1 to ARS-UCD1.2.

## Supplementary Materials

The following supporting information can be downloaded at https://www.mdpi.com/article/10.3390/ijms27219867/s1, References [32,33,34,35,36,37,38] are cited in the Supplementary Materials.

## Abbreviations

The following abbreviations are used in this manuscript:

ANOVA	Analysis of variance
BAF	B allele frequency
ChIP-seq	Chromatin Immunoprecipitation sequencing
CNV	Copy number variation
CNVR	Copy number variation region
DBSCAN	Density-Based Spatial Clustering of Applications with Noise
FDR	False Discovery Rate
FoSTeS	Fork stalling and template switching
GADA	Genome Alteration Detection Algorithm
GC	Guanine-Cytosine (content)
GWAS	Genome-wide association study
HMM	Hidden Markov model
LRR	Log R ratio
NGS	Next-generation sequencing
PCA	Principal component analysis
SNP	Single nucleotide polymorphism
TADs	Topologically associating domains
TDA	Topological data analysis
UMAP	Uniform Manifold Approximation and Projection
